# Pyroptotic cell death by exposure to 1-butanol in H9c2 cardiomyoblastoma cells

**DOI:** 10.1016/j.heliyon.2020.e05503

**Published:** 2020-11-18

**Authors:** Kanako Noritake, Toshihiko Aki, Shintaro Isa, Koichi Uemura

**Affiliations:** Department of Forensic Medicine, Graduate School of Medical and Dental Sciences, Tokyo Medical and Dental University, 113-8519, Tokyo, Japan

**Keywords:** 1-Butanol, H9c2 cells, Pyroptosis, GSDME, Caspase-3, Biochemistry, Cell biology, Immunology, Pathophysiology, Toxicology

## Abstract

The aim of this study is to examine the molecular mechanism of cytotoxicity caused by direct exposure to short chain alcohol. We showed previously that exposing H9c2 cardiomyoblastoma cells to 150 mM 1-butanol results in cell death within 1 h through an intrinsic apoptotic pathway. The cell death is accompanied by plasma membrane blebbing and caspase-3 activation. Here we show that a higher concentration (200 mM) of 1-butanol, as well as prolonged exposure (3–6 h) to 150 mM 1-butanol, induces plasma membrane ballooning, a characteristic feature of pyroptosis. Although gasderminD (GSDMD) cleavage by caspase-1 was not observed, GSDME cleavage by caspase-3 was observed during exposure to 150 mM 1-butanol for 6 h. We conclude that pyroptotic cell death by 1-butanol in H9c2 cardiomyoblastoma cells should occur via the caspase-3-GSDME pathway, revealing that 1-butanol could induce not only apoptosis but also pyroptosis in the cells.

## Teaser

1-Butanol; H9c2 cells; proptosis; GSDME; Caspase-3; Biochemistry; Cell biology; Immunology; Pathophysiology; Toxicology

## 1 Introduction

Short chain alcohols such as methanol, ethanol, propanol, and butanol are widely used in industry and the inhalation of alcohol vapor seriously affects human health. Among the detrimental effects of alcohol on human health, alcoholic cardiomyopathy sometimes has fatal impact on human health (Steiner and Lang, 2017). Death of cardiomyocytes is associated with not only cardiac pathology but also subsequent mortality. Many studies have indicated that the toxicities of short chain alcohols correlate with their hydrophobicities (Baker and Kramer, 1999). Indeed, we have shown previously that the cytotoxicities of short chain alcohols on H9c2 cardiomyoblastoma cells correlate positively with the length of the hydrocarbon chain (Noritake et al., 2012). We have also shown in a relevant study that 1-butanol induces apoptotic death in H9c2 cells. This cell death is accompanied by extensive bleb formation on the plasma membrane and is effectively suppressed by Rho-kinase inhibitor Y-27632 (Ishizaki et al., 2000), suggesting an intimate relationship among 1-butanol cytotoxicity, plasma membrane morphology, and, actomyosin contraction.

Pyroptosis is a form of cell death that is morphologically a type of necrosis, but is executed in a regulated manner (Bergsbaken et al., 2009). Although typical pyroptosis is observed in phagocytes stimulated by pro-inflammatory ligands such as lipopolysaccharides (LPS), it is also observed in a variety of cells during the administration of chemotherapy drugs (Wang et al., 2017). Pyroptosis is characterized by pore formation on the plasma membrane through which inflammatory cytokines such as interleukin-1β (IL-1β) and IL-18 are released into the extracellular milieu (Bergsbaken et al., 2009). This pore formation on the plasma membrane also results in the formation of large bubbles from the plasma membrane, which is often referred to as ballooning to distinguish it from blebbing, and subsequent cell lysis, which also contributes to the release of inflammatory cytokines from the cells.

Gasdermins (GSDMs), a family of proteins consisting of N-terminal and C-terminal domains as well as a linker region between them (Kovacs and Miao, 2017), are responsible for the pore formation on the plasma membrane during pyroptosis. Human GSDMD is cleaved after aspartate at 275, which is located in its linker region, by inflammatory caspases (caspase-1, 4, 5, and 11). The resultant N-terminal GSDMD fragment (p30) translocates to the plasma membrane where it self-oligomerizes to form pores on the plasma membrane (Kayagaki et al., 2015; Shi et al., 2015). Human GSDME is cleaved after aspartate at 270 by pro-apoptotic caspases (caspase-3 and -7) to release the pore-forming N-terminus p30 fragment (Rogers et al., 2017; Wang et al., 2017). In spite of the established roles of GSDMD and GSDME in pyroptosis, there are few reports showing the involvement of other GSDMs in pyroptotic cell death.

In this study we show that 1-butanol induces pyroptosis through caspase-3-GSDME pathway in H9c2 rat cardiomyoblastoma cells.

## 2 Materials and methods

### 2.1 Cell culture

H9c2 rat cardimyoblastoma cells were obtained from the American Type Culture Collection (ATCC, Manassas, VA) and J774.1 murine macrophage cells were obtained from RIKEN Bioresource Center (RCB0434, Tsukuba, Japan). Both cells were cultured at 37 °C in DMEM supplemented with 10% fetal bovine serum, 100 U/ml penicillin, and 100 μg/ml streptomycin. 1-Butanol (Wako Pure Chemicals, Osaka, Japan) was added to the medium at the indicated concentrations. Rho-kinase inhibitor Y-27632 (10 μM, Wako Pure Chemicals) and caspase-3 inhibitor z-DEVD-FMK (zDEVD, 10 μM, MBL, Nagoya, Japan) was added to the medium 30 min before treatment with 1-butanol. The morphology of the cells was observed under light microscopes (DMi8, Leica, Wetzlar, Germany and EVOS, Thermo Fisher Scientific, Waltham, MA).

### 2.2 Cell death assay

Cell death was assessed as LDH release into the medium using the LDH-Cytotoxic Test (Wako Pure Chemicals) according to the manufacturer's instructions.

### 2.3 Quantitative real time reverse-transcriptase-mediated PCR (qPCR)

qPCR was performed as described previously (Noritake et al., 2015; Noritake et al., 2017). In brief, total RNA was extracted from the cells using Trizol reagent (Thermo Fisher Scientific) and reverse-transcription was performed using SuperScript II (Thermo Fisher Scientific). Real time PCR was performed using StepOnePlus (Thermo Fisher Scientific) by the methods using SYBR green, and the results were quantified by the comparative Ct method. Primers used were 5′-CATCCGTTCTCTACCCAGCC-3′ and 5′-AATTCTGAGCCCGGAGTTGG-3′ for TNFα as well as 5′-GGCTCTCTGCTCCTCCCTGTTCTA-3′ and 5′-TGCCGTTGAACTTGCCGTGGG-3′ for glyceraldehyde 3-phosphate dehydrogenase (GAPDH).

### 2.4 Immunoblot analysis

For immunoblot analysis, both the cells adherent to the dishes and the cells floating in the medium were collected by low-speed centrifugation. The collected cells were lysed in lysis buffer [0.32 M sucrose, 10 mM Tris-HCl, pH 7.4, 5 mM EDTA, 50 mM NaF, 2 mM Na_3_VO_4_ and a protease inhibitor cocktail (Roche, Mannheim, Germany)], and the protein concentrations of the cell lysates were determined by the method of Bradford (1976). Equal amounts of proteins were separated by sodium dodecyl sulfate-polyacrylamide gel electrophoresis (SDS-PAGE), transferred to poly vinylidene fluoride (PVDF) membranes, and probed with following antibodies: anti-caspase-1 (ab179515, abcam, Cambridge, MA), anti-cleaved caspase-3 (#9661, Cell Signaling Technology, Beverly, CA), anti-GSDMD (sc-393656, Santa Cruz Biotechnology, Santa Cruz, CA), anti-DFNA5/GSDME (ab215191, abcam), and anti-actin (A2066, Sigma-Aldrich, St. Louis, MO). Then, antigens were visualized by use of peroxidase-conjugated anti-IgG antibodies (Promega, Madison, WI) and a Western Lightning Chemiluminescence Reagent Plus Kit (Perkin Elmer Life Science, Boston, MA).

### 2.5 Statistical analysis

All data were analyzed using the Dunnett's test and Tukey-Kramer test, and expressed as the mean ± S.D. P values < 0.05 were considered to be statistically significant.

## 3 Results

### 3.1 Time-dependent changes of cell death and plasma membrane morphology during exposure to 1-butanol

**Figure 1:**
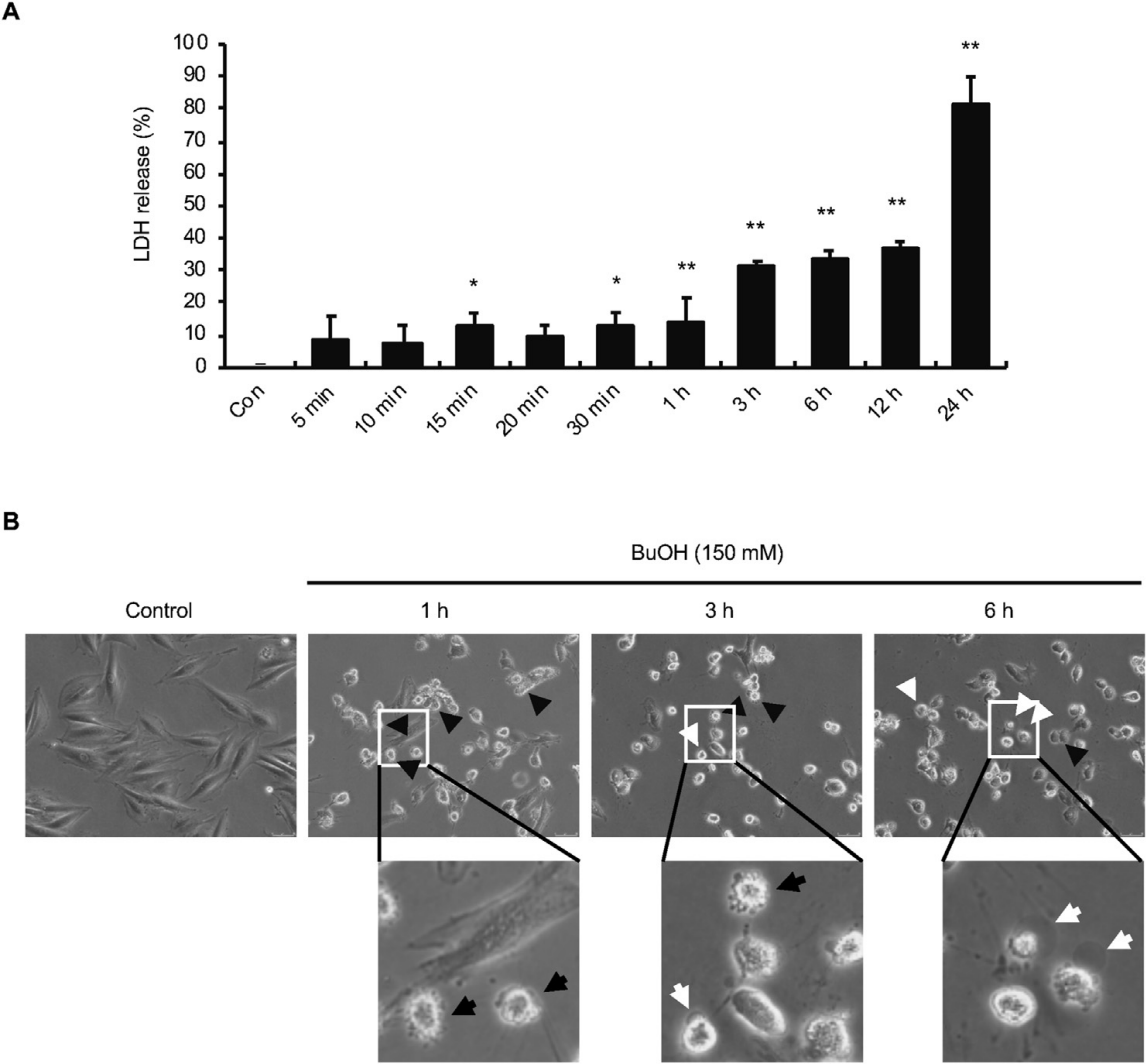
Time-dependent transition of plasma membrane morphology during exposure of H9c2 cells to 150 mM 1-butanol. (**A)** Time-dependent increase in cell death during exposure of H9c2 cells to 1-butanol. The cells were exposed to 150 mM 1-butanol for 24 h and cell death was evaluated by the LDH release assay. The graph shows mean and S.D (n = 3–8). ∗p < 0.05, ∗∗p < 0.01 versus control (Con). (**B)** Time-dependent changes in plasma membrane morphology during exposure of H9c2 cells to 1-butanol. The cells were treated with 150 mM 1-butanol for 1, 3, or 6 h and morphological changes were examined under a light microscope. Black and white arrows indicate plasma membrane blebbing and ballooning, respectively.

To confirm our previous result that exposure of H9c2 cells to 150 mM 1-butanol for 1–12 h results in a time-dependent decrease in cell viability (Noritake et al., 2012), we first performed a time course experiment assessing LDH release from cells during exposure to 150 mM 1-butanol for 24 h. As shown in Figure 1A, the percentage of LDH released from the cells into the medium increased during treatment in a time-dependent manner. Thus, concomitant with the time-dependent decrease in cell viability (Noritake et al., 2012), cell death with the loss of plasma membrane integrity increased during 150 mM 1-butanol exposure. We next evaluated the morphology of the cells during exposure to l-butanol. Close inspection of the plasma membrane morphology indicated blebbing of the plasma membrane in cells exposed to 150 mM 1-butanol for 1–3 h (Figure 1B), confirming our previous report (Noritake et al., 2012). However, further exposure to 150 mM 1-butanol resulted in the occurrence of large blebs, suggesting ballooning of the plasma membrane (Figure 1B). While plasma membrane blebbing dominated after 1 h of exposure, ballooning was more common than blebbing after 6 h of exposure (Figure 1B). These results indicate that, although cell death is induced time-dependently by 150 mM 1-butanol, the mode of cell death appears to switch as the exposure becomes more prolonged.

### 3.2 Concentration-dependent increase in cell death, plasma membrane ballooning, and TNFα expression during exposure to 1-butanol

**Figure 2:**
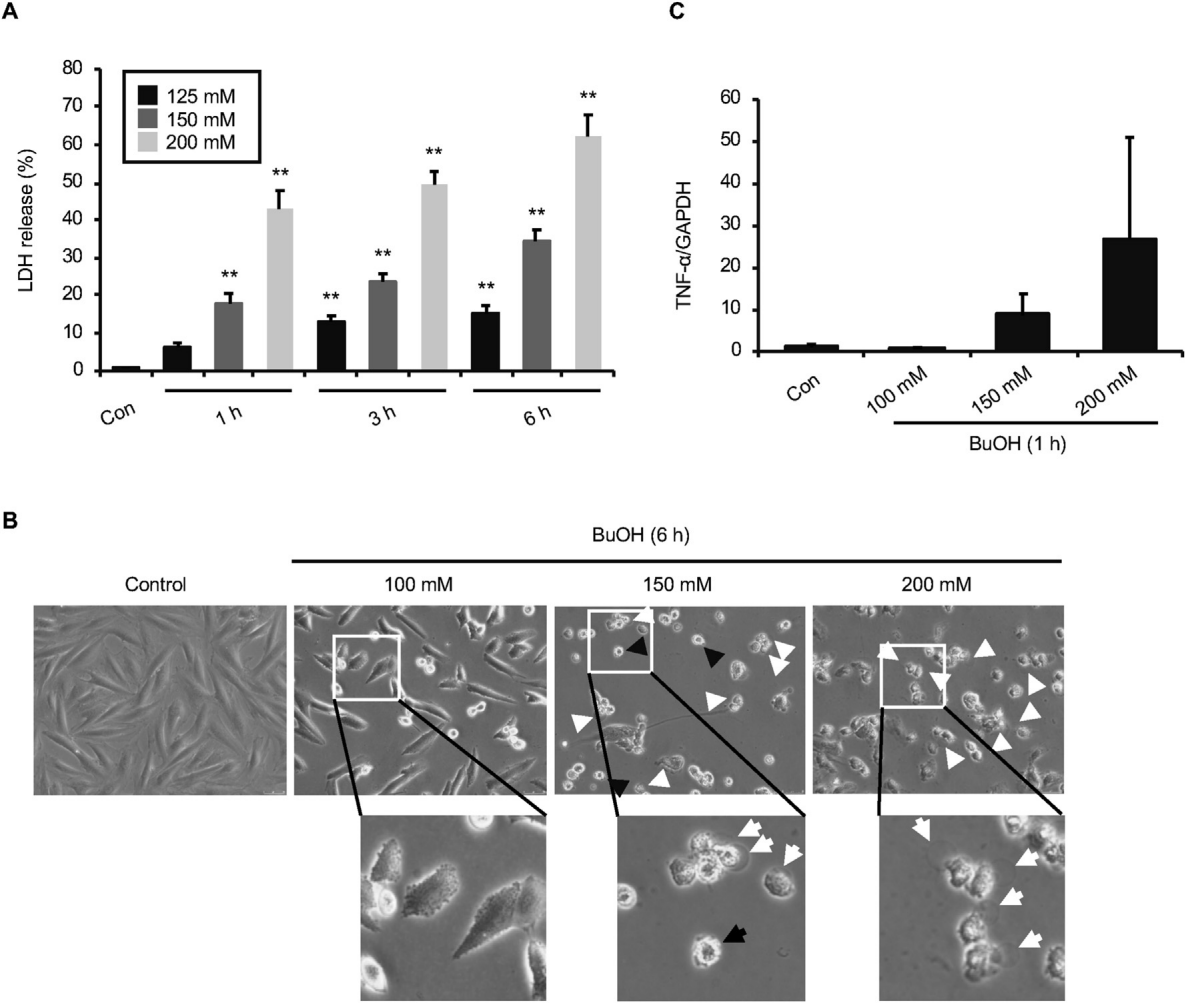
Concentration-dependent transition of plasma membrane morphology during exposure of H9c2 cells to 1-butanol. (**A)** Concentration-dependent increase in cell death during exposure of H9c2 cells to1-butanol. Cells were exposed to 125, 150 or 200 mM 1-butanol for 1, 3, or 6 h, and cell death was evaluated by the LDH release assay. The graph shows mean and S.D (n = 4–7). ∗∗p < 0.01 versus control (Con). (**B)** Concentration-dependent changes in plasma membrane morphology during exposure of H9c2 cells to 1-butanol. Cells were treated with 100, 150, or 200 mM 1-butanol for 6 h and morphological changes were examined under a light microscope. Black and white arrows indicate blebbing and ballooning, respectively. (**C)** Concentration-dependent induction of TNF-α expression during exposure of H9c2 cells to 1-butanol. Cells were exposed to 100, 150 or 200 mM 1-butanol for 1 h and the relative expression of TNF-α to GAPDH was determined by qPCR analysis. The graph shows mean ± S.D. (n = 4).

We next examined the concentration-dependency of H9c2 cell death by 1-butanol. As shown in Figure 2A, percentages of LDH release increased in a concentration-dependent manner after exposure to 125–200 mM 1-butanol for 1, 3, or 6 h. An evaluation of cell morphology after exposure to 100–200 mM 1-butanol for 6 h revealed plasma membrane ballooning overwhelming blebbing in cells exposed to 200 mM 1-butanol (Figure 2B). Since the formation of large blebs (ballooning) from the plasma membrane is one of the characteristic features of pyroptosis, a form of inflammatory cell death (Kovacs and Miao, 2017), we examined TNFα gene expression by qPCR analysis. As shown in Figure 2C, cellular TNFα gene expression showed a trend toward increasing in a concentration-dependent manner in response to exposure to 1-butanol (100–200 mM). Thus, it seems that higher concentrations of 1-butanol induce more inflammatory responses in cells. Taking the results of plasma morphology and TNFα expression together, it seems likely that the cell death that occurs during exposure to 200 mM 1-butanol is pyroptosis rather than apoptosis.

### 3.3 GSDME cleavage by caspase-3 was observed in H9c2 cells during exposure to 1-butanol

**Figure 3:**
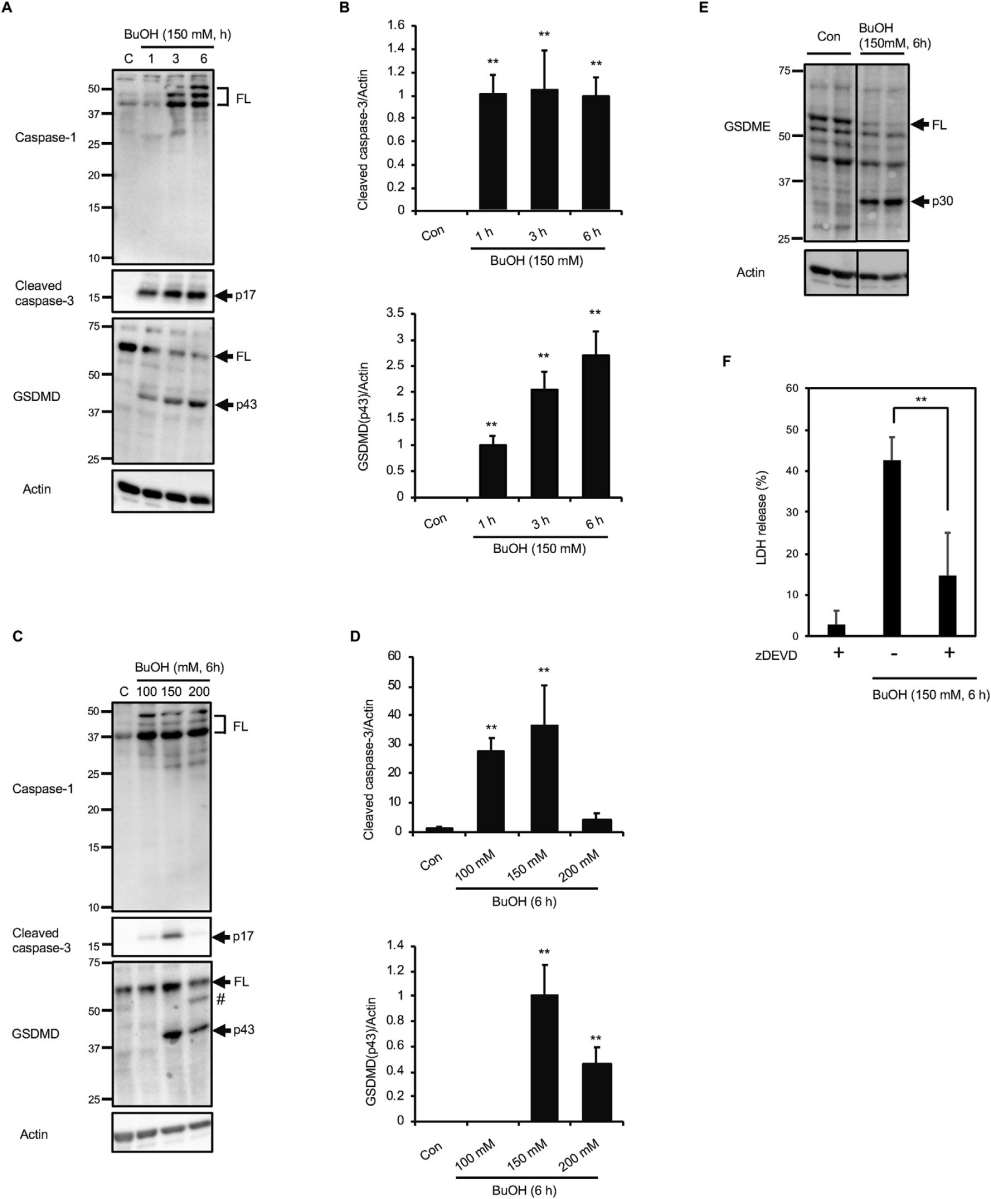
Casapse3 activation and cleavage of GSDMD and GSDME during exposure of H9c2 cells to 1-butanol. (**A** and **B)** Time-dependent activation of caspase-3 and proteolysis of GSDMD. H9c2 cells were exposed to 150 mM 1-butanol for 1–6 h, and immunoblot analyses of caspase-1, cleaved caspase-3, and GSDMD were performed. (**C** and **D)** Concentration-dependency of the activation of caspase-3 and proteolysis of GSDMD. H9c2 cells were exposed to 100–200 mM 1-butanol for 6 h, and immunoblot analyses of caspase-1, cleaved caspase-3, and GSDMD were performed. **(E)** GSDME cleavage into p30 fragment in 1-butanol-treated cells. Con, control. H9c2 cells were exposed to 150 mM 1-butanol for 6 h, and immunoblot analyses of GSDME was performed. FL indicates the full-length forms of caspase-1, GSDMD, or GSDME. #, uncharacterized fragment. The relative intensities of the bands were quantified by densitometry and normalized to actin. The graph shows mean ± S.D. (n = 4). ∗∗p < 0.01 versus control (Con). (**F**) Cells were exposed to 150 mM 1-butanol for 6 h in the absence or presence of 10 μM zDEVD and LDH release were assessed. The graph shows mean ± S.D. (n = 3). ∗∗p < 0.01.

There are at least two established mechanisms for the ballooning from the plasma membrane during pyroptosis: GSDMD cleavage by inflammatory caspases such as caspase-1 (Kayagaki et al., 2015; Shi et al., 2015), and GSDME cleavage by caspase-3 (Rogers et al., 2017; Wang et al., 2017). We, therefore, examined the time-dependent activation of caspase-1 and -3, as well as the cleavages of GSDMD, in H9c2 cells exposed to 150 mM 1-butanol for 1–6 h. Immunoblot analysis of caspase-1 revealed several bands corresponding to the molecular weights of full-length inactive precursor forms at around 45 kDa (Figure 3A). The detection of multiple bands is probably due to alternative splicing of this gene (Alnemri et al., 1995). Although the abundance of these precursor bands seemed to be upregulated during exposure to 150 mM 1-butanol for 1–6 h, the active p10 and p12 forms were not observed (Figure 3A). Cleaved (activated) caspase-3 was observed as early as 1 h after exposure to 150 mM 1-butanol (Figure 3 A and B), consistent with our previous results (Noritake et al., 2012). In agreement with the lack of caspase-1 activation, the p30 fragment of GSDMD was not observed (Figure 3A). Instead, a p43 fragment, which is the product of caspase-3-dependent cleavage of GSDMD after Asp87, and which does not have pore-forming activity (Taabazuing et al., 2017), was observed (Figure 3A). The time-dependency of the formation of the p43 GSDMD fragment correlates well with the generation of cleaved-caspase-3 (p17) (Figure 3 A and B), confirming that p43 should indeed be the product of caspase-3-dependent cleavage of GSDMD. We next examined the concentration-dependent activations of caspase-1 and -3, as well as the cleavages of GSDMD. Similar to the time-dependent increase observed during exposure to 150 mM 1-butanol, the full-length forms of caspase-1 seemed to be upregulated by 1-butanol (Figure 3C). However, neither the p10 nor p12 active form was detected at any concentration (100, 150, and 200 mM) of 1-butanol (Figure 3C). Also, the p43 fragment was observed instead of p30 in the immunoblot analysis of GSDMD (Figure 3C). In contrast to the results for the time course experiments, the levels of caspase-3 p17 as well as GSDMD p43 peaked at 150 mM 1-butanol (Figure 3 C and D). We examined GSDME in the cells exposed to 150 mM 1-butanol for 6 h and found that p30 fragment GSDME was observed in the cells (Figure 3E). We also observed that the caspase-3 inhibitor zDEVD reduced LDH release from 1-butanol treated cells significantly but not completely (Figure 3F). These results indicate that caspase-3-dependent cleavage of GSDME is partially responsible for the pyroptotic cell death during 1-butanol exposure.

### 3.4 Activation of caspase-3/GSDME pathway was also observed in J774.1 cells during exposure to 1-butanol

**Figure 4:**
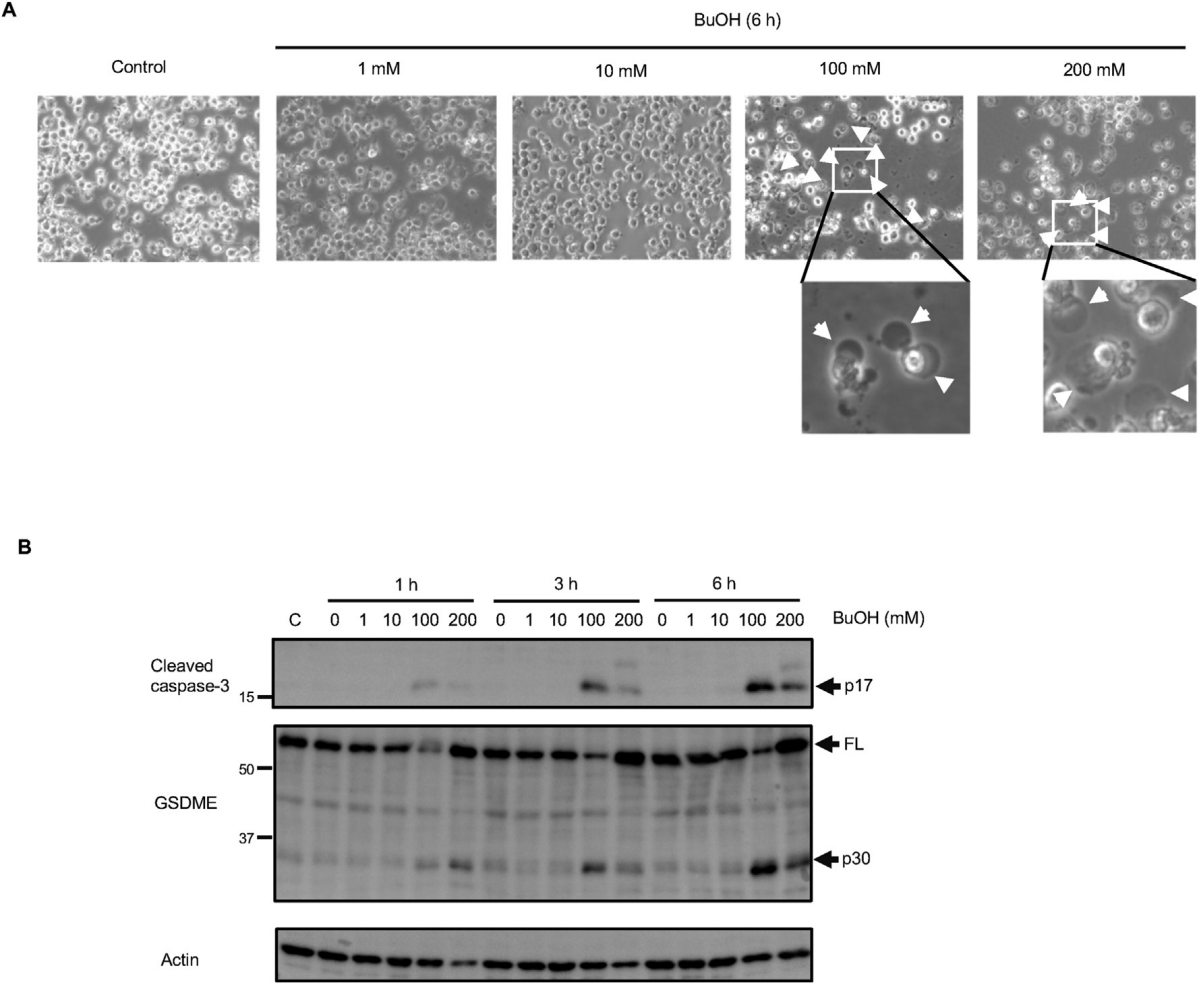
Casapse3 activation and cleavage of GSDME during exposure of J774.1 cells to 1-butanol. (**A)** Concentration-dependent changes in plasma membrane morphology during exposure of J774.1 cells to 1-butanol. Cells were treated with 1, 10, 100 or 200 mM 1-butanol for 6 h and morphological changes were examined under a light microscope. White arrows indicate ballooning. (**B)** Time-dependent activation of caspase-3 and proteolysis of GSDME. J774.1 cells were exposed to 1, 10, 100 or 200 mM 1-butanol for 1, 3 and 6 h, and immunoblot analyses of cleaved caspase-3 and GSDME were performed. C, control. FL indicates the full-length forms of GSDME.

We further examined whether GSDME cleavage by caspase-3 upon exposure to 1-butanol is specific to H9c2 or not. J774.1 murine macrophage cells were used for this purpose. J774.1 cells were exposed to gradually increased concentrations of 1-butanol (1, 10, 100, and 200 mM). Plasma membrane ballooning was observed in the cells treated with 100 and 200 mM 1-butanol for 6 h (Figure 4A). Immunoblot analysis showed that caspase-3 p17 as well as GSDME p30 was observed in the cells after 1–6 h exposure to 100–200 mM 1-butanol (Figure 4B). These results indicate that pyroptosis through caspase-3/GSDME pathway by 1-butanol is not specific to H9c2 cells.

### 3.5 Y-27632 suppresses pyroptosis

**Figure 5:**
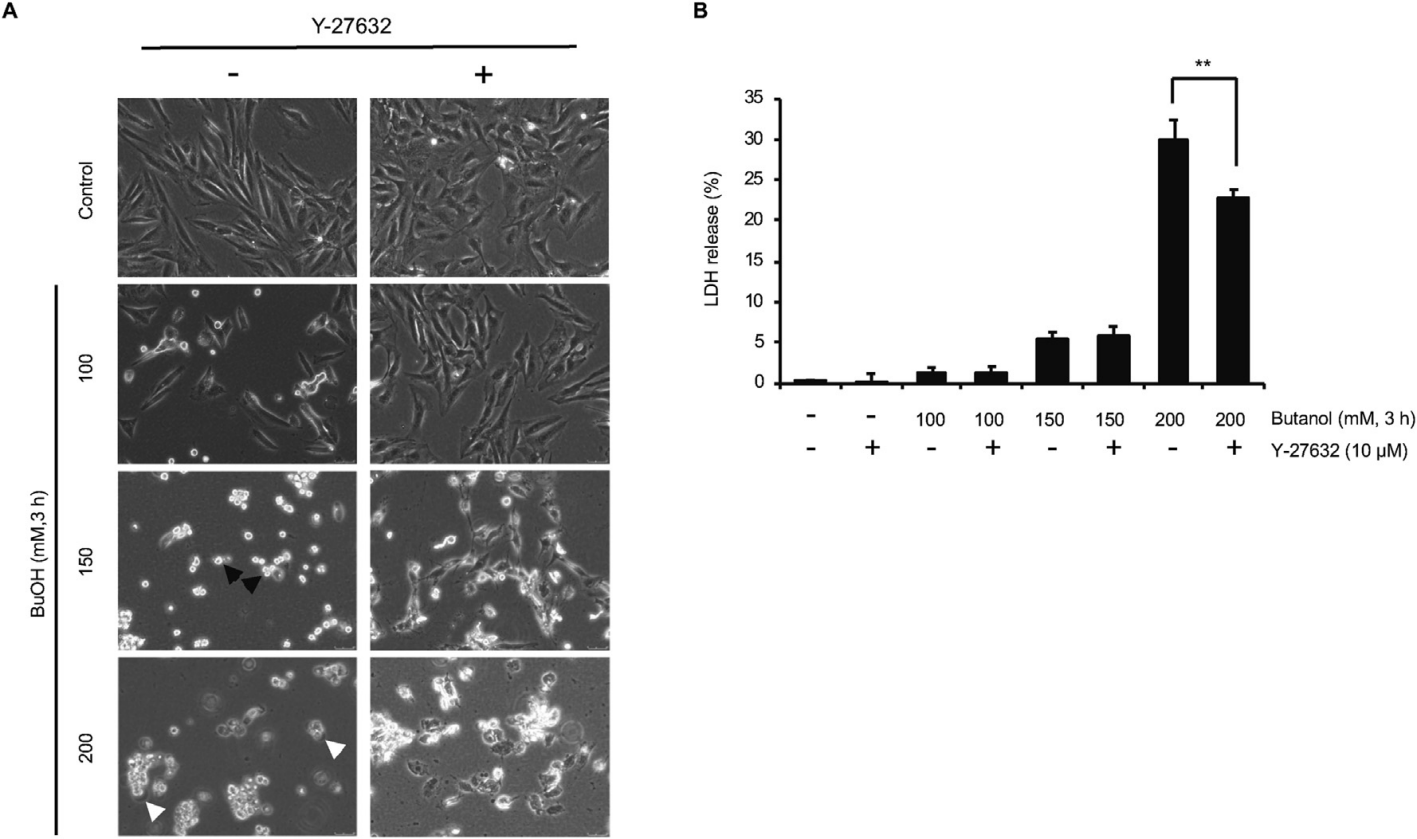
Effect of Y-27632 on cell morphology as well as LDH release in H9c2 cells exposed to 1-butanol. (**A** and **B)** Cells were exposed to 100, 150, or 200 mM 1-butanol for 3 h in the absence or presence of 10 μM Y-27632, and cell morphology (**A**) and LDH release (**B**) were assessed. Black and white arrows indicate blebbing and ballooning, respectively. ∗∗p < 0.01 versus control.

We examined the effects of Y-27632 on apoptotic as well as pyroptotic cell death by 1-butanol. Plasma membrane blebbing of the cells by 150 mM 1-butanol seemed to be ameliorated by Y-27632 (Figure 5A), as we have shown previously (Noritake et al., 2012). Interestingly, Y-27632 also ameliorated the ballooning by 200 mM 1-butanol (Figure 5A). Y-27632 had no effect on LDH release from cells treated with 150 mM 1-butanol (Figure 5B). In contrast, Y-27632 significantly suppressed LDH release from cells treated with 200 mM 1-butanol (Figure 5B). Taken together, it seems that Rho-kinase is involved in ballooning as well as the pyroptotic cell death by 1-butanol.

## 4 Discussion

It has been reported that short chain alcohols, including 1-butanol, suppress NLRP3 inflammasomes when applied at 0.38–3% to the J774 murine macrophage cell line (Hoyt et al., 2016). Since 150 and 200 mM 1-butanol correspond to 1.37 and 1.83%, respectively, the 1-butanol concentrations used in this study should inhibit NLRP3 inflammasomes, which are required for caspase-1 activation as well as for the synthesis of mature IL-1β and IL-18. The lack of caspase-1 activation in 1-butanol-exposed cells (Figure 3) might be, therefore, due to the inhibitory effect of 1-butanol on NLRP3 inflammasomes. The caspase-3-dependent cleavage of GSDME accounts for much of the pyroptosis caused by chemotherapy drugs (Wang et al., 2017), and this is also the case for the pyroptosis of H9c2 cells by 1-butanol.

We also show the possible involvement of Rho-kinase in plasma membrane ballooning as well as pyroptotic cell death (Figure 5). Plasma membrane blebbing is an active cellular process that results from increased actomyosin activity and resultant detachment of the plasma membrane from the actin cytoskeleton (Charras, 2008). In contrast, ballooning is considered to be passive swelling of cells. The formation of GSDM pores on the plasma membrane leads to a disruption in the concentration gradients of sodium as well as potassium ions across membranes. The subsequent influx of extracellular fluids through GSDM pores results in ballooning and the subsequent rupture of the plasma membrane (Kovacs and Miao, 2017). Indeed, it has been demonstrated that Rho-kinase and actomyosin contraction are not involved in plasma membrane ballooning during GSDMD-mediated pyroptosis (de Vasconcelos et al., 2019). However, our current results (Figure 5) indicate the possible involvement of Rho-kinase in pyroptosis under certain situations.

Time-dependent transition from apoptosis to necrosis is a frequently observed phenomenon and well-known as secondary necrosis. In contrast, there are not many examples of concentration-dependent switching from apoptosis to necrosis. Within 100–200 mM 1-butanol, LDH release from H9c2 cells increased in a concentration-dependent manner (Figure 2A). However, cleavage of caspase-3 into p17 was peaked at 150 mM 1-butanol (Fig 3C and D). Since pyroptotic necrosis through GSDME is mediated by caspase-3, unknown mechanism other than caspase-3/GSDME might be responsible for the increased pyroptotic necrosis in H9c2 cells treated with 200 mM 1-butanol. Indeed, zDEVD could not completely suppress cell death by 150 mM 1-butanol (Figure 3F). Further study should be necessary to elucidate the transition mechanism of cell death mode observed around 150 mM 1-butanol in H9c2 cells. In conclusion, we showed that 1-butanol induces pyroptosis through caspase-3-GSDME pathway in H9c2 cells. These results reveal the importance of pyroptosis in the cytotoxicity of alcohol.

## Declarations

### Author contribution statement

K. Noritake: Conceived and designed the experiments; Performed the experiments.

A. Toshihiko: Conceived and designed the experiments; Wrote the paper.

S. Isa: Performed the experiments.

K. Uemura: Analyzed and interpreted the data.

### Funding statement

K. Noritake was supported by Grant-in Aid for Scientific Research from the 10.13039/501100001700Ministry of Education, Culture, Sports, Sciences and Technology of Japan (19K19483). T. Aki was supported by Grant-in Aid for Scientific Research from the 10.13039/501100001700Ministry of Education, Culture, Sports, Sciences and Technology of Japan (18K19670).

### Declaration of interests statement

The authors declare no conflict of interest.

### Additional information

No additional information is available for this paper.
